# Correlation between uterine microbiota and pregnancy outcomes of embryo transfer in overweight and obese women

**DOI:** 10.3389/fcimb.2025.1515563

**Published:** 2025-02-03

**Authors:** Ying Yan, Ying Li, Lingling Wu, Yuxuan Zhang, Hong Guo, Yazhong Ji

**Affiliations:** Reproductive Medical Center, Tongji Hospital, School of Medicine, Tongji University, Shanghai, China

**Keywords:** infertility, uterus, microbiota, obesity, RNA Ribosomal 16S

## Abstract

**Objective:**

Currently, it has been reported that uterine microbiota affected pregnancy outcomes in assisted reproductive technology, but little was known in obese women. Thus, this study aims to explore how body weight affects pregnancy outcomes by comparing the differences in uterine microbiota between women of normal weight and those with obesity or overweight.

**Methods:**

The study included 45 embryo transfer cycles. Patients were divided into two groups based on body mass index (BMI): normal-weight group, BMI ≤23.9 kg/m² (Con group, n=31) and obesity/overweight group, BMI >23.9 kg/m² (OwOb group, n=14). Microbial samples were collected from the catheter tips and analyzed using RNA Ribosomal 16S.

**Results:**

In 45 women, the β-human chorionic gonadotropin (β-hCG) positivity rate and clinical pregnancy rate 10-12 days after embryo transfer were higher in the Con group. At the genus level, the relative abundance of Lactobacillus in the Con group was 2.2 times higher than that in the OwOb group. The Alpha diversity in the OwOb group was significantly higher than in the Con group (p=0.028). After regrouping based on β-hCG results 10-12 days post-transfer, in the Con-negative group (the group with negative β-hCG in the Con group, n=16) the relative abundances of pathogenic bacteria such as Klebsiella (p<0.001), Parasutterella (p=0.004), Dialister (p=0.01), and Gardnerella (p=0.029) were significantly higher than in the Con-positive group (the group with positive β-hCG in the Con group, n=15).

**Conclusion:**

Overweight and obese women possess a distinct uterine microbiota, characterized by a lower relative abundance of Lactobacillus and an increased relative abundance of pathogenic bacteria, along with specific genera strongly associated with obesity. In individuals with higher BMI, pathogenic bacteria are more likely to impair endometrial receptivity, ultimately leading to adverse pregnancy outcomes. Therefore, modulating the uterine microbiota in overweight/obese women may improve assisted reproductive technology success rates.

## 1 Introduction

Obesity is closely linked to female infertility. In China, a body mass index (BMI) greater than 23.9 kg/m² is classified as overweight, and a BMI above 27.9 kg/m² is classified as obese. The prevalence of overweight and obesity is increasing globally. A substantial body of research indicates a negative correlation between the success rate of assisted reproductive technologies (ART) and weight gain (Kalliala et al., 2017; Amiri and Ramezani Tehrani, 2020; Cozzolino et al., 2021). Weight gain can adversely affect female reproductive capacity through various mechanisms, including hormonal imbalances, chronic inflammation, immune dysfunction, psychological factors, and particularly changes in the microbiota, which is an emerging research focuses in the recent years (Gautam et al., 2023).

The role of microbial communities in female reproductive health is increasingly recognized. Although only about 10% of the human body’s microbiota resides in the urogenital system, these microorganisms play a critical role in maintaining the health and stability of both the urinary and reproductive systems (Günther et al., 2022). The advent of next-generation sequencing (NGS) technologies enabled researchers to detect uterine microbiota by targeting the variable regions of the 16S rRNA gene (Mitchell et al., 2015; Cicinelli et al., 2015). These findings have been corroborated by multiple sampling methods, which have confirmed the presence of a minimal but distinct uterine microbiota (Mitchell et al., 2015; Editorial Office, 2021; Vomstein et al., 2022). Furthermore, significant differences between the microbiota of the upper and lower reproductive tracts have been identified (Carosso et al., 2020; Vitale et al., 2021), providing strong evidence for the existence of a unique uterine microbiota.

Current studies have reached a consensus on the composition of the uterine microbiota. At the genus level, Lactobacillus is the dominant genus, with other commonly detected genera including *Prevotella, Bifidobacterium, Corynebacterium, Gardnerella, Haemophilus, Propionibacterium, Staphylococcus*, and *Streptococcus*. The composition of the uterine microbiota has been shown to be associated with pregnancy outcomes, particularly following embryo transfer, where a higher abundance of Lactobacillus correlates positively with implantation rates (Moreno et al., 2022). In contrast, patients with recurrent implantation failure (RIF) and recurrent spontaneous abortion (RSA) have been found to have a lower abundance of Lactobacillus compared to controls (Chen et al., 2022; Liu et al., 2022). Additionally, there is a negative correlation between Lactobacillus and pathogenic bacteria such as Prevotella, Gardnerella, Streptococcus, and Klebsiella, suggesting that Lactobacillus may play a crucial role in maintaining the stability of the uterine ecosystem (Chen et al., 2022).

Studies have demonstrated that weight gain is associated with reduced diversity in gut microbiota and an increased ratio of Firmicutes to Bacteroidetes in the intestines of obese individuals (Turnbaugh et al., 2008; Le Chatelier et al., 2013). Altered gut microbiota in overweight and obese populations can disrupt digestion and metabolism by inducing low-grade inflammation, ultimately contributing to disease (Marchesi et al., 2016). Similarly, overweight women undergoing ART are found to have lower oocyte retrieval rates, fewer high-quality embryos, and reduced clinical pregnancy rates, alongside higher rates of biochemical pregnancy and miscarriage (Zhang et al., 2017; Rafael et al., 2023). Recent studies have found that the infertile Obese women are more likely to have endometritis due to the presence of pathogenic bacteria in the endometrium (King et al., 2024).

Building on these findings, the present study aims to compare the uterine microbiota of overweight/obese infertile women with that of normal-weight infertile women to identify differences, which could help pinpoint uterine microbial characteristics strongly associated with body weight. This research could inform targeted health management strategies for infertile women and provide a theoretical foundation for potential therapeutic interventions.

## 2 Materials and methods

### 2.1 Study population

This study enrolled 45 infertile women who underwent *in vitro* fertilization and embryo transfer (IVF-ET) at the Reproductive Medicine Center of Tongji Hospital in Shanghai between September 2022 and February 2023. The inclusion criteria were as follows: women aged 21 to 45 years, and infertility caused by tubal factors was confirmed (confirmed by hysterosalpingography), and the total number of antral follicles ≥8, currently undergoing an embryo freeze-thaw transfer cycle, negative serological tests for hepatitis B virus (HBV), hepatitis C virus (HCV), and human immunodeficiency virus (HIV), and not currently pregnant or breastfeeding.

Exclusion criteria were: diagnosis of polycystic ovary syndrome, premature ovarian insufficiency, acute or chronic reproductive tract inflammation, human papillomavirus (HPV) infection, chronic diseases (such as diabetes, hypertension, cardiovascular and cerebrovascular diseases), chromosomal abnormalities, or endometrial conditions (such as adenomyosis, submucosal or intramural fibroids > 4 cm, endometrial cancer, or uterine septum). Additional exclusions included male factor infertility, recurrent implantation failure, unexplained infertility, use of oral antibiotics within one month prior to the transfer, invasive procedures within one month, sexual intercourse within 48 hours before the transfer, and absence of viable embryos available for transfer.

### 2.2 Data collection

Data on the general characteristics of the study participants were recorded, including age, BMI on the day of transfer, baseline hormone levels, duration of infertility, type of infertility, reproductive history, number of previous transfers, number and type of embryos transferred, and hormone levels on the day of endometrial transformation.

### 2.3 Group assignment

A total of 45 infertile women were enrolled in this study. Participants were grouped based on their BMI, measured on the morning of the transfer day. Those with a BMI ≤23.9 kg/m² were classified into the normal-weight group (Con, n=31), while those with a BMI >23.9 kg/m² were categorized into the overweight/obese group (OwOb, n=14). The original two groups (Con and OwOb groups) were further subdivided based on β-hCG results 10-12 days post-embryo transfer into four subgroups: normal-weight β-hCG positive (Con-positive, n=15), normal-weight β-hCG negative (Con-negative, n=16), overweight/obese β-hCG positive (OwOb-positive, n=5), and overweight/obese β-hCG negative (OwOb-negative, n=9). Please refer to the **Supplementary Material** for a detailed endometrial treatment regimen.

### 2.4 Sample collection

Participants provided informed consent before the procedure. On the embryo transfer day, each patient was placed in the lithotomy position. A sterile speculum was inserted to visualize the cervix, followed by cleansing of the vaginal and cervical areas three times using sterile cotton swabs moistened with 37°C saline. Under transabdominal Doppler ultrasound guidance (Philips, EPIQ7), a sterile double-sheath transfer catheter (Cook, G24216) was carefully introduced into the uterine cavity through the cervical canal without contacting the vaginal walls, ensuring that the inner sheath remained fully enclosed within the outer sheath during the insertion. Once the catheter reached the internal cervical os, the tip of the inner sheath containing the embryo was gently extended beyond the outer sheath and positioned 1-2 cm from the uterine fundus. After a 30-second dwell, the inner sheath was retracted 10 mm into the outer sheath, and the catheter was cautiously removed as a unit, avoiding any contact with the vaginal walls. After holding the catheter with the tip downward and flushing the outer sheath with sterile saline, the anterior 5 mm of the outer sheath was then cut off, and the inner sheath was re-extended. The inner sheath was then flushed with 100 µL of sterile phosphate-buffered saline (Bioagrio, LS2041-500), and the sample was immediately stored at -80°C for further analysis.

### 2.5 DNA extraction and amplification

Total genomic DNA was extracted using MagPure Soil DNA LQ Kit (Magan, D6356-02) following the manufacturer’s instructions. DNA concentration and integrity were measured with NanoDrop 2000 (Thermo Fisher Scientific, USA) and agarose gel electrophoresis. Extracted DNA was stored at -20°C until further processing. The extracted DNA was used as template for PCR amplification of bacterial 16S rRNA genes with the barcoded primers and Takara Ex Taq (Takara). For bacterial diversity analysis, V3-V4 (or V4-V5) variable regions of 16S rRNA genes was amplified with universal primers 343F (5’-TACGGRAGGCAGCAG-3’) and 798R (5’-AGGGTATCTAATCCT-3’) for V3-V4 regions.

### 2.6 Library construction and sequencing

The Amplicon quality was visualized using agarose gel electrophoresis. The PCR products purified with AMPure XP beads (Agencourt) and amplified for another round of PCR. After purified with the AMPure XP beads again, the final amplicon was quantified using Qubit dsDNA Assay Kit (Life Technologies,Q32854). The concentrations were then adjusted for sequencing. Sequencing was performed on an Illumina NovaSeq 6000 with 250 bp paired-end reads.

### 2.7 Data analysis

All sequence data were processed using QIIME2 software (Bolyen et al., 2019), with default parameters for quality filtering, denoising, merging, and chimera removal. Operational taxonomic units (OTUs) and amplicon sequence variants (ASVs) were generated, and representative sequences were annotated against the SILVA database. Alpha diversity including ACE, P-whole-tree, Chao1, Shannon index and Beta diversity including unweighted and weighted Unifrac principal coordinates analysis (PCoA) were analyzed using QIIME2. Differential analysis between groups was conducted using R statistical software, employing ANOVA, Kruskal-Wallis, t-test, or Wilcoxon tests. The LEfSe method was used for differential species analysis.

The rest of the statistical analysis was conducted using SPSS 26.0 software. Independent sample T-tests were used for normally distributed data, while Mann-Whitney U tests were employed for non-normally distributed data. The Wilcoxon rank-sum test or chi-square test was applied to compare differences between groups. The Kruskal-Wallis test was used for multiple group comparisons, and Pearson correlation was performed for correlation analysis. Fisher’s exact test was used when any expected count was less than 5. *P*-value of less than 0.05 was considered statistically significant.

## 3 Results

### 3.1 Comparison of patient baseline characteristics

This study included 45 infertile women, grouped into a normal-weight group (Con, n=31; BMI ≤23.9 kg/m²) and an overweight/obese group (OwOb, n=14; BMI >23.9 kg/m²). The basic demographic information of the participants is summarized in **Table 1**. The average BMI was significantly different between the Con group (21.07 ± 1.67 kg/m²) and the OwOb group (26.67 ± 2.11 kg/m²) (*P* < 0.001). There were also significant differences in estradiol (E_2_) levels measured on the day of endometrial transformation (median: Con group = 0.71 nmol/L, OwOb group = 1.98 nmol/L, *P* = 0.032). However, other demographic characteristics, such as age, baseline anti-Müllerian hormone (AMH), baseline follicle-stimulating hormone/luteinizing hormone (FSH/LH), duration of infertility, type of infertility, number of previous embryo transfers, Endometrial thickness, progesterone and LH levels on the day of endometrial transformation, and endometrial preparation protocol during the transfer cycle, did not differ significantly between the two groups (**Table 1**).

**Table 1:** Demographic characteristics of study participants.

	Con group (n = 31)	OwOb group (n = 14)	*P* value
Body mass index (kg/m^2^)	21.07 ± 1.67	26.67 ± 2.11	< 0.001***
Age (years)	34.61 ± 5.70	35.36 ± 6.58	0.701
History of previous embryo transfers	1 (0,2)	0 (0,2)	0.515
Duration of infertility (years)	3.0 (2.0,5.0)	4.5 (2.0,6.3)	0.520
AMH (ng/ml)	2.48 (1.22,3.90)	3.64 (2.18,5.95)	0.198
Basic FSH/LH	1.52 (0.94,2.67)	1.38 (0.88,2.22)	0.590
Endometrial Transformative Day Hormone Levels			
P (nmol/L)	20.55 ± 16.14	16.78 ± 12.00	0.440
E_2_ (nmol/L)	0.71 (0.45,2.53)	1.98 (1.39,4.34)	0.032*
LH (U/L)	3.68 (0.99,7.05)	2.66 (0.57,11.13)	0.840
FSH (IU/L)	2.87 (1.71,5.96)	2.19 (1.10,3.93)	0.257
No. of embryos transferred	1 (1,2)	1 (1,2)	0.795
Endometrial thickness(mm)	9.0 (8.27,10.92)	9.4 (8.25,10.65)	0.750
Infertility type			0.654
Primary infertility (%)	9 (29.0%)	5 (35.7%)	
Secondary Infertility (%)	22 (71.0%)	9 (64.3%)	
Endometrial preparation protocols for embryo transfer			0.456
Natural cycle (%)	3 (9.7%)	2 (14.3%)	
Hormone replacement cycle (%)	25 (80.6%)	12 (85.7%)	
Ovarian stimulation cycle (%)	3 (9.7%)	-	

*AMH*, anti-müllerian hormone; *FSH*, follicle-stimulating hormone; *LH*, luteinizing hormone; *P*, progestin; *E_2_*, estradiol. Asterisks denote the difference is significant between groups (*P < 0.05, ***P < 0.001). Quantitative data with normal distribution and equal variance were analyzed using the independent two-sample t-test. For data that did not meet the normality assumption or had unequal variances, the Mann-Whitney U test was used. Qualitative data were analyzed using the chi-square test.

During a 14 week follow-up period, clinical pregnancy outcomes were monitored. 10-12 days after embryo transfer, serum β-human chorionic gonadotropin (β-hCG) levels were measured, showing 20 positive cases (44.4%) and 25 negative cases (55.6%) among the 45 women. Of the 20 positive cases, 4 experienced biochemical pregnancy loss and 1 had an early miscarriage. By the 14th week of gestation, 15 women were ongoing pregnancy, with 2 experiencing late pregnancy loss. Ultimately, 13 women successfully delivered live births (**Table 2**).

**Table 2:** Differences in pregnancy outcomes between two groups of patients.

	Con group (n = 31)	OwOb group (n = 14)	*P* value
Embryo implantation rate (%)	34.9 (15/43)	15.0 (3/20)	0.125
Biochemical pregnancy rate (%)	48.4 (15/31)	35.7 (5/14)	0.525
Clinical pregnancy rate (%)	41.9 (13/31)	21.4 (3/14)	0.313
Biochemical pregnancy loss rate (%)	13.3 (2/15)	40.0 (2/5)	0.249
Ongoing pregnancy rate (%)	38.7 (12/31)	21.4 (3/14)	0.321
Live brith rate (%)	35.5 (11/31)	14.3 (2/14)	0.178

The chi-square test was used to analyze the data.

Data analysis showed embryo implantation rate (Con vs. OwOb: 34.9% vs. 15.0%, *P* = 0.123), biochemical pregnancy rate (Con vs. OwOb: 48.4% vs. 35.7%, *P* = 0.525), clinical pregnancy rate (Con vs. OwOb: 41.9% vs. 21.4%, *P* = 0.313), ongoing pregnancy rate (Con vs. OwOb: 38.7% vs. 21.4%, *P* = 0.321), and live brith rate (Con vs. OwOb: 35.5% vs. 14.3%, *P* = 0.178)in the Con group were higher than those in the OwOb group, while biochemical pregnancy loss rate was higher in the OwOb group than in the Con group (Con vs. OwOb: 13.3% vs. 40.0%, *P* = 0.249). Due to the small sample size, none of these differences reached statistical significance (**Table 2**).

### 3.2 Uterine microbiota distribution

**Figure 1:**
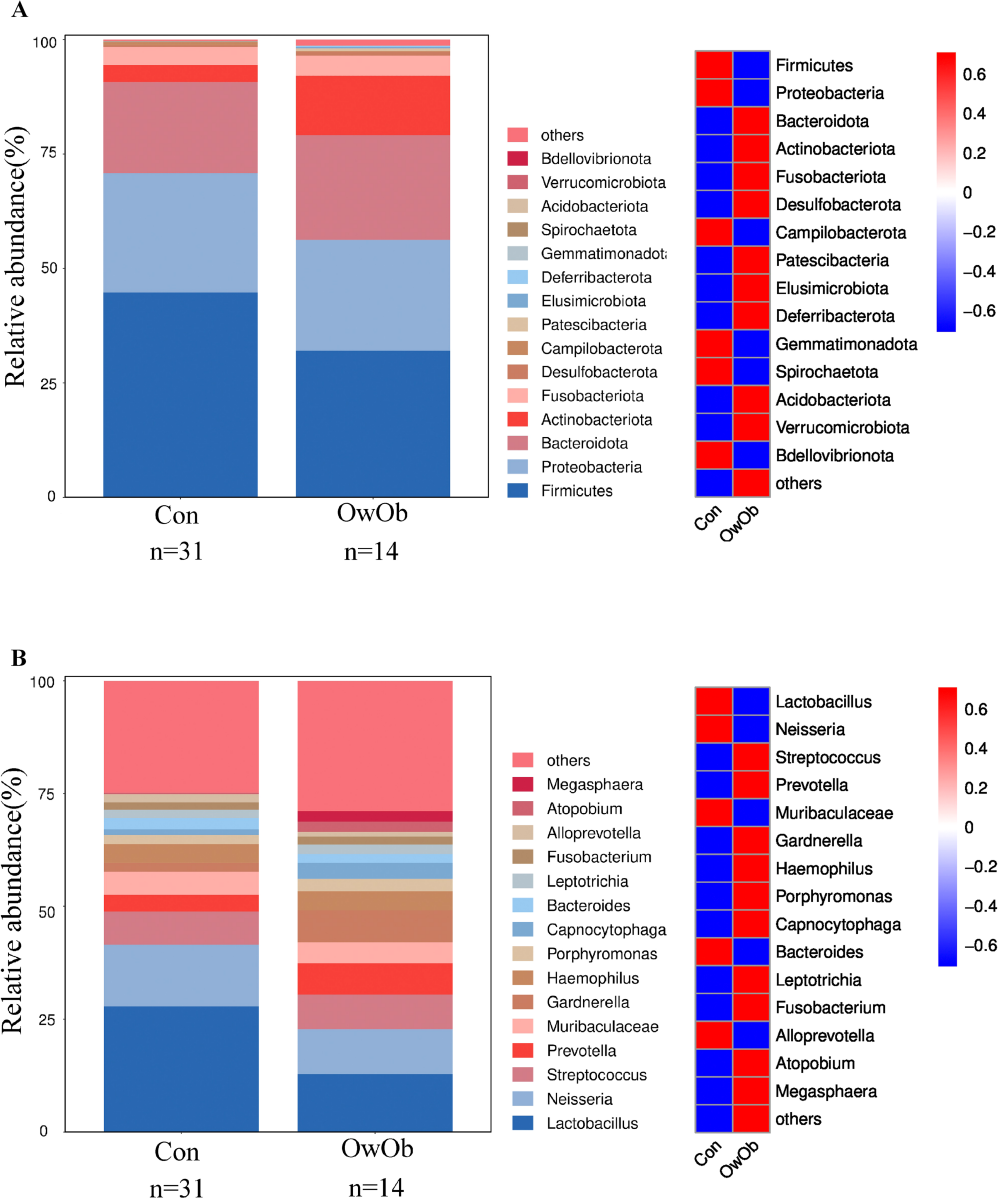
Display the distribution of microbial taxa between groups. **(A)** Bar graph (left) and heatmap (right) show the relative distribution of the top fifteen dominant phyla in groups. Group name: Con = normal-weight group (n = 31), OwOb = overweight/obese group (n = 14). **(B)** Bar graph (left) and heatmap (right) show the relative distribution of the top fifteen dominant genera in groups. Group name: Con = normal-weight group (n = 31), OwOb = overweight/obese group (n = 14).

16S rRNA sequencing detected microbiota in endometrial tissue samples collected from all 45 women. The relative distribution of the top 15 most dominant species at the phylum level has been shown (**Figure 1A**). Firmicutes and Proteobacteria were the predominant phylum in both the Con and OwOb groups, followed by Bacteroidota, Actinobacteriota, Fusobacteriota, Desulfobacterota, Campilobacterota and Patescibacteria. The most statistically significant difference at the phylum level was for Actinobacteriota (*P* = 0.002). Patescibacteria had a higher relative abundance in the OwOb group, which was nearly absent in the Con group (*P* = 0.009). The relative abundance of Firmicutes was higher in the Con group, though not statistically significant (*P* = 0.127). There were no significant differences between the groups in the relative abundance of Proteobacteria, Bacteroidota, or Fusobacteriota (**Supplementary Table S1**).

At the genus level, Lactobacillus was the dominant genus in both the Con and OwOb groups. However, the relative abundance of Lactobacillus in the Con group was approximately 2.2 times higher than in the OwOb group, although this difference was not statistically significant (*P* = 0.133) (**Supplementary Table S2**). The relative abundances of Streptococcus, Prevotella, Gardnerella, Haemophilus, Porphyromonas, Capnocytophaga and Atopobium were higher in the OwOb group compared to the Con group (**Figure 1B**). Notably, the relative abundance of Capnocytophaga was significantly greater in the OwOb group (*P* = 0.012). Additionally, Parasutterella was found at very low levels, nearly absent, in the Con group but present in small amounts in the OwOb group, with the difference between the groups reaching statistical significance (*P* = 0.025) (**Supplementary Table S2**).

### 3.3 Diversity of uterine microbiota between groups

**Figure 2:**
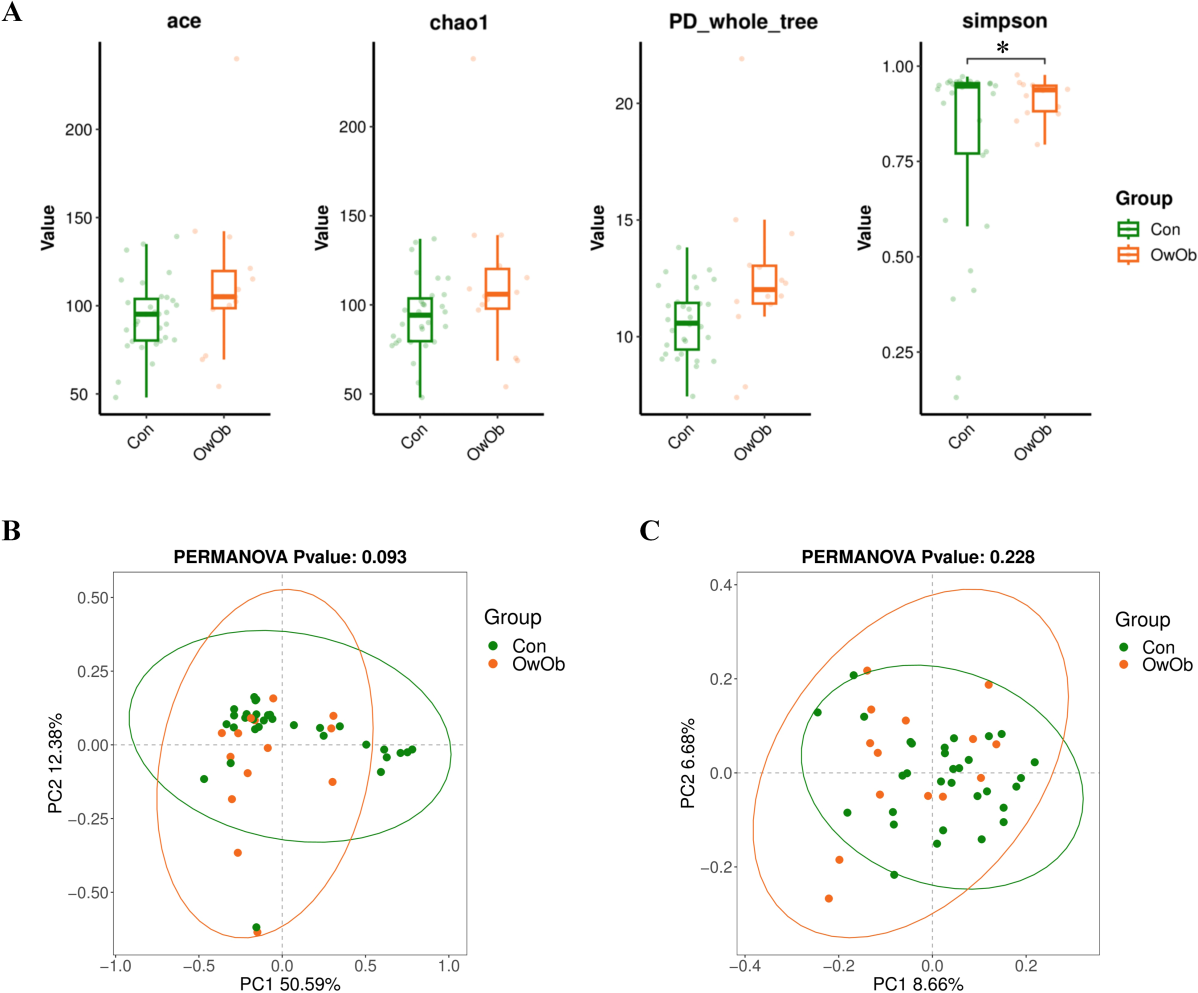
**(A)** Boxplots comparing alpha diversity index between the Con and OwOb groups (Con = normal-weight group, OwOb = overweight/obese group), calculated using the ACE (*P* = 0.16), Chao1 (*P* = 0.16), PD-whole-tree (*P* = 0.16), and Simpson (*P* = 0.028) index. Con group (n = 31), OwOb group (n = 14). *P < 0.05. **(B)** PCoA analysis of beta diversity index comparing the Con and OwOb groups, with confidence ellipses based on the Weighted Unifrac distance algorithm (*P* = 0.093). Con group (n = 31), OwOb group (n = 14). **(C)** PCoA analysis of beta diversity index comparing the Con and OwOb groups, with confidence ellipses based on the Unweighted Unifrac distance algorithm (*P* = 0.228). Group name: Con group (n = 31), OwOb group (n = 14).

To investigate intra-group and inter-group diversity of the endometrial microbiota, we performed both Alpha and Beta diversity analyses. Alpha diversity assesses the diversity within a single sample group, reflecting species richness and evenness. In this study, Alpha diversity was calculated using the ACE index (*P* = 0.16), P_whole_tree index (*P* = 0.076), Chao1 index (*P* = 0.16), and Simpson index (*P* = 0.028). Box plots constructed for these indices indicate that intra-group diversity in the OwOb group was higher than in the Con group. Notably, the Simpson index demonstrated a significantly greater intra-group microbial diversity in the OwOb group compared to the Con group (*P* = 0.028) (**Figure 2A**).

Beta diversity analysis was conducted to compare the microbial community composition between the two groups. This analysis used PCoA based on a distance matrix calculated from species abundance data. The weighted Unifrac (**Figure 2B**) and UnWeighted Unifrac (**Figure 2C**) methods were employed. The Weighted Unifrac analysis, which considers both presence and abundance of species, showed a moderate difference between the two groups (*P* < 0.1); (**Figure 2C**).

### 3.4 Analysis of uterine microbiota composition differences between groups

**Figure 3:**
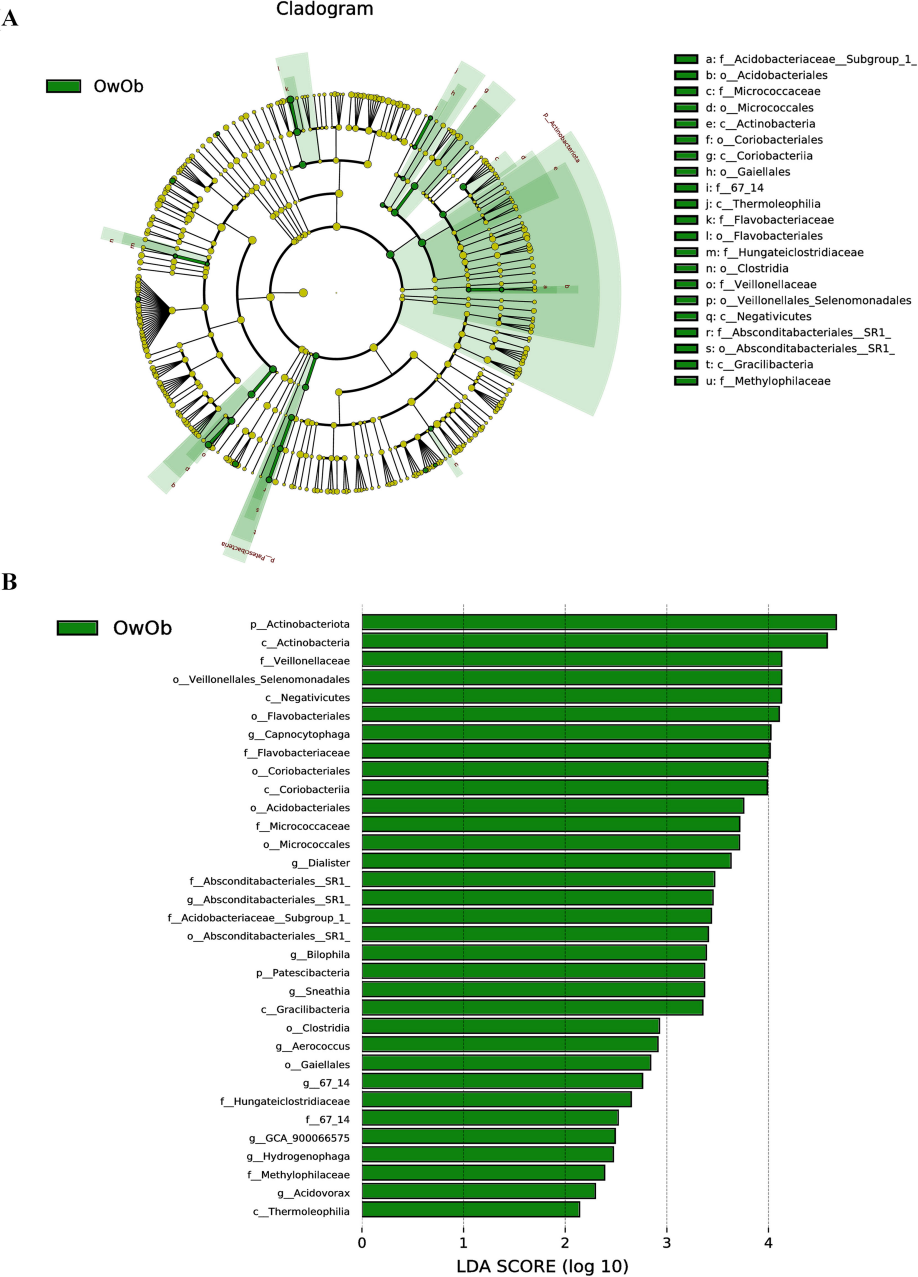
Linear discriminant analysis coupled with effect size measurements (LEfse) allows comparisons between groups and look for Biomarkers that are statistically different between groups. **(A)** Circles radiating from inner to outer ring represent taxonomic levels from phylum to genus. Each small circle at a different taxonomic level represents a taxon at that level, and the size of the circle diameter is proportional to the relative abundance. Light green nodes indicate significantly different species with relatively high abundance in the OwOb (overweight/obese group, n = 14) group, yellow nodes indicate species that are not significantly different in the comparison of the two groups, and the size of the node diameter is proportional to the size of the relative abundance. No species were found to be significantly different in the Con group (normal-weight group, n = 31). **(B)** Bar graphs show biomakers with statistically significant differences in LDA scores > 2. Light green bars show species with significant differences in abundance in the OwOb group (n = 14), with the length of the bars representing the effect size for significantly different species.

To identify biomarkers with statistically significant differences between the two groups, this study employed Linear Discriminant Analysis Effect Size (LEfSe) analysis, which is a robust tool that detects high-dimensional biomarkers and provides insight into whether specific microbial communities are uniquely present in different groups. A greater number of biomarkers were identified in the OwOb group. At the phylum level, Actinobacteriota and Patescibacteria were characteristic of the OwOb group. At the genus level, the OwOb group was distinguished by specific microbial taxa, including Capnocytophaga, Dialister, Absconditabacteriales-SR1, Bilophila, and Sneathia (**Figures 3A, B**).

### 3.5 Comparative analysis of microbiota in subgroups with different implantation outcomes

**Figure 4:**
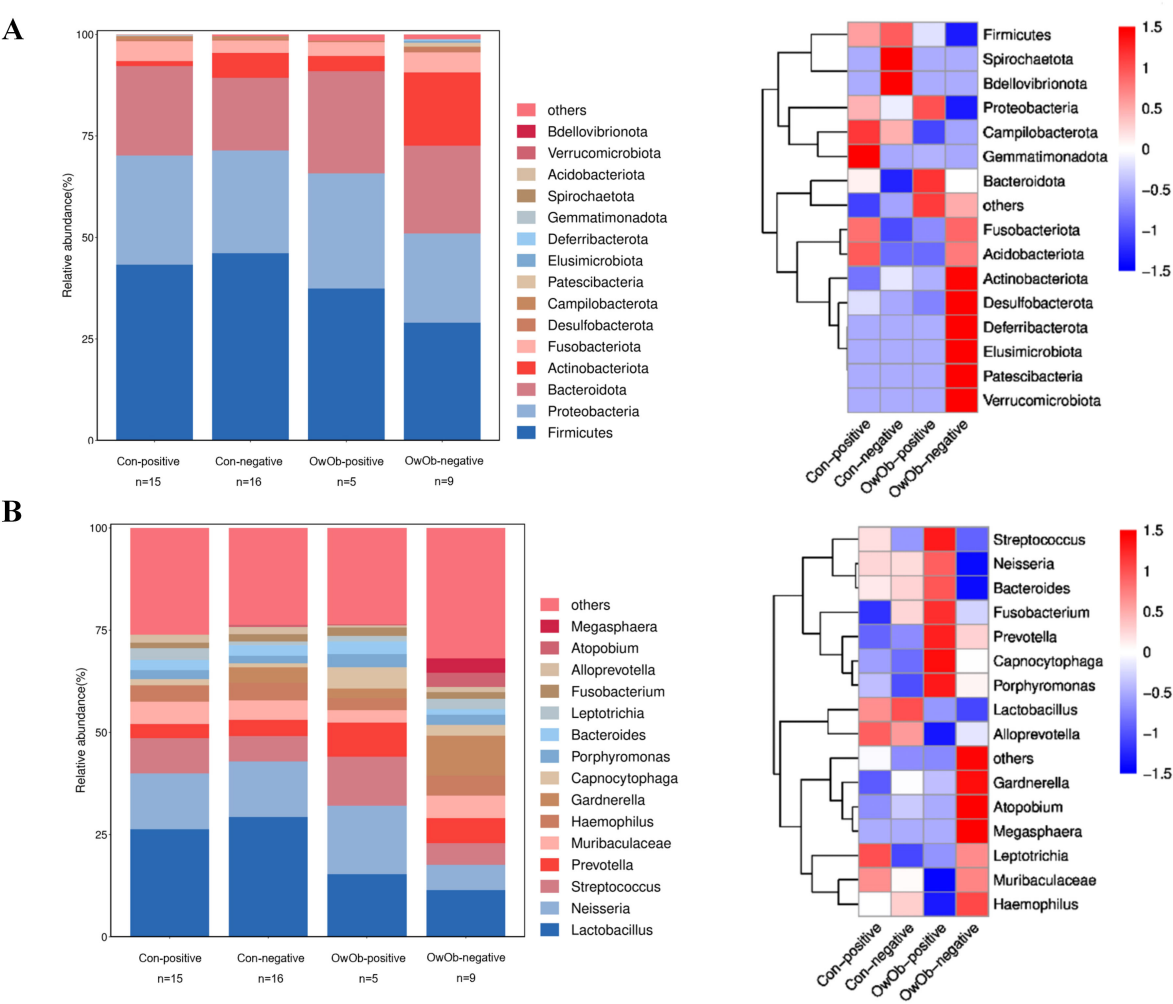
Displays subgroup microbiota profiles and differences in specific genera between groups. The original two groups were subdivided based on β-hCG results 10-12 days post-embryo transfer into four subgroups: Con-positive = normal-weight β-hCG positive group (n = 15), Con-negative = normal-weight β-hCG negative group (n = 16), OwOb-positive = overweight/obese β-hCG positive group (n = 5), and OwOb-negative = overweight/obese β-hCG negative group (n = 9). **(A)** Bar plot (left) and heatmap (right) showing the relative abundance of bacterial phyla across four groups. **(B)** Bar plot (left) and heatmap (right) illustrating the relative abundance of bacterial genera across four groups.

The original two groups (Con and OwOb groups) were further subdivided based on β-hCG results 10-12 days post-embryo transfer into four subgroups: normal-weight β-hCG positive (Con-positive, n=15), normal-weight β-hCG negative (Con-negative, n=16), overweight/obese β-hCG positive (OwOb-positive, n=5), and overweight/obese β-hCG negative (OwOb-negative, n=9). Data were re-analyzed for these subgroups (**Figures 4A, B**).

At the genus level, Lactobacillus remained the most abundant genus in all groups, followed by Neisseria, Streptococcus, Prevotella, and others (**Figure 4B**). The relative abundance of Lactobacillus was highest in the Con-negative group and lowest in the OwOb-negative group. Specific genera were showed significant differences among the groups. Gardnerella had the highest relative abundance in the OwOb-negative group (0.098), followed by the Con-negative (0.038), OwOb-positive (0.024), and Con-positive (<0.001) groups (**Table 3**). Other genera with significant intergroup differences included Dialister, Bergeyella, Parasutterella, Klebsiella, and Leptotrichia. Klebsiella demonstrated the most pronounced difference across the groups (*P*=0.001), with trace amounts detected in the OwOb-negative group (**Table 3**).

**Table 3:** Relative abundance of genera at the level of bacterial genera in four groups of patients.

Genus	Con-positive group (n = 15)	Con-negative group (n = 16)	OwOb-positive group (n = 5)	OwOb-negative group (n = 9)	*P* value
*Gardnerella*	1.6×10^-4^	3.8×10^-2^	2.4×10^-2^	9.8×10^-2^	0.028*
*Dialister*	0	3.3×10^-3^	1.0×10^-2^	9.8×10^-3^	0.024*
*Bergeyella*	3.0×10^-2^	2.8×10^-4^	1.6×10^-2^	2.5×10^-4^	0.011*
*Parasutterella*	9.3×10^-6^	1.4×10^-4^	1.7×10^-3^	1.0×10^-2^	0.039*
*Klebsiella*	0	9.7×10^-4^	2.8×10^-5^	8.3×10^-3^	0.001**
*Sneathia*	0	0	0	7.6×10^-3^	0.042*

Asterisks denote the difference is significant between groups (*P < 0.05, **P < 0.01).

Fisher’s exact probability method was used to analyze the data.

**Figure 5:**
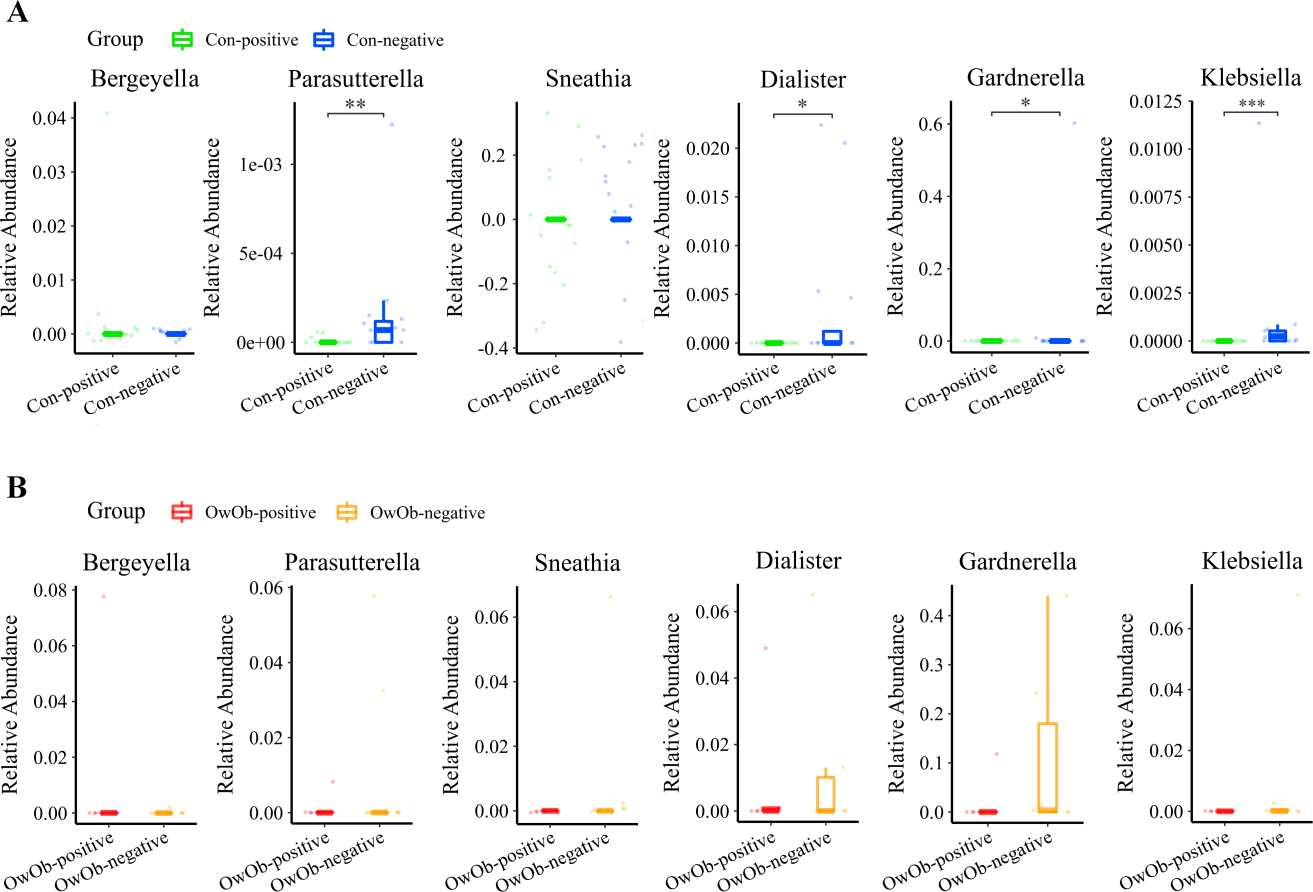
**(A)** Boxplot comparing the differential abundance of specific genera between the Con-positive (n = 15) and Con-negative groups (n = 16) (Bergeyella, *P* = 0.150; Parasutterella, *P* = 0.004; Sneathia, *P* = 1.000; Dialister, *P* = 0.011; Gardnerella, *P* = 0.029 and Klebsiella, *P* < 0.001). The relative abundance of Parasutterella, Dialister, Gardnerella and Klebsiella was significantly higher in the Con-negative group compared to the Con-positive group. **(B)** Boxplot comparing the differential abundance of specific genera between the OwOb-positive (n = 5) and OwOb-negative groups (n = 9) (Bergeyella, *P* = 0.095; Parasutterella, *P* = 0.570; Sneathia, *P* = 0.320; Dialister, *P* = 0.890; Gardnerella, *P* = 0.150 and Klebsiella, *P* = 0.250). Parasutterella, Sneathia, Dialister, Gardnerella and Klebsiella showed higher relative abundances in the OwOb-negative group compared to the OwOb-positive group, although these differences were not statistically significant. (Con-positive = normal-weight β-hCG positive group, n = 15; Con-negative = normal-weight β-hCG negative group, n = 16; OwOb-positive = overweight/obese β-hCG positive group, n = 5; OwOb-negative = overweight/obese β-hCG negative group, n = 9.) *P < 0.05, **P < 0.01 and ***P < 0.001. The Wilcoxon test was used for the inter-group comparisons involved in this figure.

The differences in specific genera between the Con-positive group and the Con-negative group were compared. The results indicate that Klebsiella (*P* < 0.001), Parasutterella (*P* = 0.004), Dialister (*P* = 0.011), and Gardnerella (*P* = 0.029) exhibited significant statistical differences between the two groups, with higher relative abundances in the Con-negative group (**Figure 5A**). These genera were nearly undetectable in the Con-positive group.

A similar comparison was conducted between the OwOb-positive group and OwOb-negative group within the overweight and obese women. As depicted in the box plot Gardnerella and Dialister showed higher relative abundances in the OwOb-negative group compared to the OwOb-positive group. However, the differences showed no statistical significant (**Figure 5B**).

## 4 Discussion

In this study, we focused on infertile patients who were overweight or obese (OwOb) and examined the differences in uterine microbiota between these individuals and those of normal weight using 16S rRNA gene sequencing. The findings revealed that Lactobacillus was the predominant genus in both groups. However, the relative abundance of Lactobacillus was significantly lower in the OwOb group compared to the normal-weight group. Additionally, intra-group microbial diversity was notably higher in the OwOb patients, suggesting a more diverse and varied microbial composition in their endometrial microbiota. This increased diversity may indicate a disrupted microbial environment in the uterus, although the differences in species diversity between the two groups did not reach statistical significance. These results suggest that obesity may alter the uterine microbial ecosystem, potentially affecting reproductive outcomes.

Our findings suggest trends of clinical results consistent with previous research, indicating that women with higher BMI may face challenges in achieving pregnancy. However, the observed differences did not reach statistical significance due to the limited sample size.

Our findings align with the broader literature on microbiota composition. Studies consistently report that, at the phylum level, Firmicutes is the most abundant, followed by *Proteobacteria, Bacteroidota*, and *Actinobacteriota* in endometrial microbiota (Moreno et al., 2016; Pelzer et al., 2018; Sun et al., 2021). Additional research indicates that certain genera, such as Atopobium, Bifidobacterium, Gardnerella, and Streptococcus—all of which are linked to adverse pregnancy outcomes—are more prevalent in overweight/obese women (Diaz-Martínez et al., 2021; Chen et al., 2022). In our study, we observed higher relative abundances of Streptococcus, Gardnerella, and Atopobium in the OwOb group compared to the Con group. Correspondingly, OwOb group have relatively poor pregnancy outcomes.

It is now widely accepted that adverse pregnancy outcomes is linked to a reduction in Lactobacillus abundance (Moreno et al., 2016). The presence of Lactobacillus in the uterine cavity plays a critical role in inhibiting the growth of pathogenic bacteria (Melgaço et al., 2018). Women with a Lactobacillus-dominant microbiota have higher implantation rate, pregnancy rate, and live birth rate after IVF-ET compared to those who do not dominate the microbiota (Peuranpää et al., 2022; Su et al., 2024). Conversely, other studies suggest that achieving such high levels of Lactobacillus in the uterine cavity may not be common (Leoni et al., 2019; Moreno et al., 2022; Reschini et al., 2022). Even with meticulous aseptic sampling techniques designed to avoid contamination from vaginal and cervical flora, cases of Lactobacillus dominance are infrequent, with Lactobacillus accounting for only 7% of samples collected via hysteroscopy, likely due to potential contamination from the lower reproductive tract microbiota (Vitale et al., 2021). Our findings show that normal-weight women had a higher relative abundance of Lactobacillus (27.9%) compared to overweight/obese women (12.6%). However, samples with >90% Lactobacillus abundance were rare in both groups.

Previous studies on the vaginal microbiota of overweight versus normal-weight women have shown that body weight is positively correlated with microbial diversity and Prevotella abundance (Raglan et al., 2021), but negatively correlated with Lactobacillus abundance (Allen et al., 2022). A large cross-sectional study of 6,000 participants found a higher incidence of bacterial vaginosis in overweight/obese women (Brookheart et al., 2019). Our study extends these findings to the endometrial microbiota, reinforcing the link between body weight and microbial diversity. Of particular note is Parasutterella, a genus not commonly mentioned in uterine microbiota studies but identified in gut microbiota research, where it is found in higher abundance in obese and type 2 diabetes patients. Reduced carbohydrate intake in obese individuals has been associated with decreased Parasutterella abundance, suggesting the presence of a dietary carbohydrate-microbiota-host metabolic axis (Henneke et al., 2022). In our study, Parasutterella abundance was significantly higher in the endometrial microbiota of overweight/obese patients compared to normal-weight women, hinting at a similar metabolic axis within the uterus.

Regarding microbiota diversity, our findings align with the majority of previous studies, revealing higher α-diversity in the uterine microbiota of infertile women in the OwOb group. This suggests that obesity, as a suboptimal health condition, contributes to increased complexity in the uterine microbiota. Previous studies have also observed elevated α-diversity in the uterine microbiota of patients with recurrent implantation failure (Liu et al., 2022) or chronic endometritis (Zhang et al., 2024). Based on these observations, we propose that higher α-diversity may serve as a predictor of poor endometrial receptivity, potentially indicating an increased risk for adverse reproductive outcomes.

To further understand the relationship between microbiota and implantation outcomes, we regrouped patients based on successful or failed implantation and analyzed their microbiota composition. We found that the relative abundance of Lactobacillus was slightly higher in normal-weight women who experienced failed implantation compared to those with successful implantation. Both groups, however, had higher Lactobacillus levels than overweight/obese women. Conversely, overweight/obese women with failed implantation had higher relative abundances of genera such as Klebsiella, Parasutterella, Gardnerella, and Leptotrichia compared to the other groups. Among normal-weight women, those with failed implantation exhibited significantly higher abundances of Klebsiella, Parasutterella, Dialister, and Gardnerella compared to those who successfully implanted. These genera are known to be associated with various gynecological conditions, suggesting that the presence of pathogenic bacteria in the uterine cavity may negatively impact pregnancy outcomes. Furthermore, obesity may facilitate the colonization of these pathogens, further disrupting the uterine environment. In normal-weight women, implantation failure was associated with significantly higher relative abundances of four specific bacterial genera compared to those with successful implantation. In contrast, although certain genera, such as Gardnerella and Dialister, showed higher relative abundances in the implantation failure subgroup of overweight/obese women, these differences were not statistically significant. This result deviates somewhat from our initial expectations, primarily due to the small sample size in each subgroup after further division of the OwOb group, which limited our ability to detect statistically significant differences. However, the overall trend observed is encouraging and should be taken into consideration.

Current research suggests that the endometrial microbiota may influence endometrial receptivity by modulating the local immune microenvironment (Lee et al., 2024). Commensal bacteria stimulate endometrial cells to produce antimicrobial substances and mucins, forming a protective barrier. Additionally, they secrete metabolites like polysaccharides and short-chain fatty acids, creating a microenvironment that deters pathogens. These bacteria also enhance immune cells’ recognition of pathogen-associated molecular patterns, collectively supporting embryo implantation by modulating the local immune environment and promoting endometrial receptivity (Al-Nasiry et al., 2020). In cases of endometrial dysbiosis, levels of inflammatory cytokines, such as IL-6, IL-1β, HIF-1α, and COX-2, are significantly elevated in endometrial fluid, while anti-inflammatory factors like IL-10 and IGF-1 are markedly reduced (Cela et al., 2022). This inflammatory state may further promote pathogenic bacterial invasion, leading to pathological changes in the endometrium and creating a vicious cycle (Al-Nasiry et al., 2020). Therefore, identifying therapeutic strategies to restore the balance of the uterine microbiota could potentially improve pregnancy rates in infertile patients.

The longstanding neglect of the uterine microbiota may contribute to the persistently low endometrial receptivity in some infertile women, hindering successful pregnancy outcomes (Simón, 2022). In addition to causing implantation failure, low endometrial receptivity can negatively impact maternal health during pregnancy, increasing the risk of complications such as preeclampsia, placental abruption, and premature rupture of membranes (Benner et al., 2018). Numerous studies have demonstrated that microbial balance can improve pregnancy outcomes. Lactoferrin administration improved the uterine microbiota in 43.2% of women with recurrent implantation failure, increasing live birth rates and enhancing endometrial receptivity (Kitaya and Ishikawa, 2022). Furthermore, co-culturing Lactobacillus rhamnosus BPL005 with pathogens reduced the abundance of pathogens and significantly decreased inflammatory markers (Chenoll et al., 2019), suggesting the potential therapeutic role of probiotics or prebiotics in some infertile women (Schenk et al., 2021). In our study, we observed that the relative abundances of pathogenic genera, such as Gardnerella, Klebsiella, and Atopobium, were significantly higher in overweight/obese women. These genera are known to be associated with an increased risk of adverse reproductive outcomes. Given these findings, strategies aimed at correcting uterine microbiota dysbiosis in overweight/obese women may offer a promising therapeutic approach to improve ART outcomes. One such approach could involve probiotic supplementation to restore beneficial Lactobacillus levels, which are critical for maintaining a healthy uterine environment. Additionally, antibiotic treatment targeting excess pathogenic genera like *Gardnerella* and *Klebsiella* could help restore microbial balance and improve endometrial receptivity. For overweight women with a history of implantation failure, uterine microbiota profiling could be employed to assess their microbial composition, allowing for the development of personalized treatment strategies. Such tailored interventions may help restore microbial balance, ultimately enhancing implantation success and reducing the risk of miscarriage in overweight and obese women undergoing assisted reproductive technology.

However, there are limitations to this study, including the small sample size. Previous studies have consistently shown that patients with a high BMI have lower pregnancy rates than normal-weight patients (Sun et al., 2020; Schenk et al., 2021), but the limited sample size in this study prevented us from detecting significant differences in pregnancy outcomes between the groups. It should be acknowledged that the extensive exclusion criteria applied in this study may lead to selection bias, as patients with more complex infertility causes were excluded. Therefore, the results may not fully represent the broader population of women undergoing assisted reproductive technology, particularly those with more severe or multifactorial infertility issues. Additionally, there is a potential for contamination of endometrial fluid by cervical and vaginal microbiota, despite our strict adherence to double-sheath catheter sampling techniques. Future studies could involve sampling endometrial, cervical, and vaginal fluids from the same patient for a cross-sectional comparison to confirm the purity of the uterine sample before inclusion in the analysis. Lastly, there was a statistically significant difference in endometrial estrogen levels on the endometrial transformative day between the two groups. Elevated estrogen may negatively affect embryo implantation (Ma et al., 2003), potentially influencing pregnancy outcome data in this study. Patients with higher body weights generally receive higher doses of estrogen during hormone replacement cycles. To address this, future studies should either increase sample size or include patients with more uniform baseline data to reduce statistical bias.

The strength of this study lies in its novel focus on comparing the uterine microbiota between overweight/obese and normal-weight patients, aiming to identify specific bacterial communities that may affect embryo implantation. The discovery of the uterine microbiota may open new avenues for diagnosing and treating infertility related to endometrial receptivity. Future research could focus on interventions to modulate the uterine microenvironment, aiming to identify bacteria strongly associated with infertility. Additional studies should explore changes in the uterine microbiota before and after treatment in infertile women to further substantiate the link between the microbiota and infertility. We further hypothesize that successful weight loss in these patients could improve pregnancy outcomes by altering the microbiota. Investigating the pathophysiological mechanisms by which the uterine microbiota influences embryo implantation, placental formation, and fetal development could also provide valuable insights.

In conclusion, based on these findings, we propose that there are differences in the endometrial microbiota between overweight/obese and normal-weight women. Overweight/obese women are more likely to have an unfavorable microbiota, with a decreased abundance of Lactobacillus, which may affect endometrial receptivity through a diet-microbe-host metabolic axis, ultimately impacting pregnancy outcomes. Our study provides a novel perspective on why pregnancy rates in women with high BMI are generally lower than those in normal-weight women.

## Data Availability

The data presented in the study are deposited in the BioProject repository, accession number: BioProject ID: PRJNA1206777.
