# Supplementary material for: Correlation between uterine microbiota and pregnancy outcomes of embryo transfer in overweight and obese women
Source: Front Cell Infect Microbiol. 2025 Feb 3;15:1515563. doi: 10.3389/fcimb.2025.1515563 (PMC11830673; doi:10.3389/fcimb.2025.1515563)
Supplement: Supplementary file 1 [file DataSheet1.docx]

**Table S1.** Relative abundance of each phylum at the bacterial phylum level in the Con and OwOb groups

| Phylum | Con group (n = 31) | OwOb group (n = 14) | *P* value |
| --- | --- | --- | --- |
| Firmicutes | 0.448 | 0.320 | 0.127 |
| Proteobacteria | 0.261 | 0.242 | 0.781 |
| Bacteroidota | 0.199 | 0.229 | 0.362 |
| Actinobacteriota | 0.038 | 0.129 | 0.002** |
| Fusobacteriota | 0.039 | 0.044 | 0.526 |
| Desulfobacterota | 0.005 | 0.009 | 0.354 |
| Campilobacteria | 0.006 | 0.001 | 0.485 |
| Patescibacteria | ＜0.001 | 0.006 | 0.009** |

Asterisks denote the difference is significant between groups (*P* < 0.05 and **P < 0.01).

Mann-Whitney U Test was used to analyze the data.
