# Supplementary material for: Correlation between uterine microbiota and pregnancy outcomes of embryo transfer in overweight and obese women
Source: Front Cell Infect Microbiol. 2025 Feb 3;15:1515563. doi: 10.3389/fcimb.2025.1515563 (PMC11830673; doi:10.3389/fcimb.2025.1515563)
Supplement: Supplementary file 2 [file DataSheet2.docx]

**Table S2.** Relative abundance of each genera at the bacterial genera level in the Con and OwOb groups

| genera | Con group (n = 31) | OwOb group (n = 14) | *P* value |
| --- | --- | --- | --- |
| Lactobacillus | 0.279 | 0.128 | 0.133 |
| Neisseria | 0.136 | 0.099 | 0.313 |
| Streptococcus | 0.074 | 0.077 | 0.781 |
| Prevotella | 0.037 | 0.069 | 0.088 |
| Muribaculaceae | 0.051 | 0.046 | 0.337 |
| Gardnerella | 0.020 | 0.071 | 0.172 |
| Haemophilus | 0.041 | 0.042 | 0.759 |
| Capnocytophaga | 0.012 | 0.036 | 0.012* |
| Porphyromonas | 0.020 | 0.028 | 0.279 |
| Bacteroides | 0.025 | 0.020 | 0.362 |
| Leptotrichia | 0.019 | 0.021 | 0.339 |
| Fusobacterium | 0.016 | 0.018 | 0.611 |
| Atopobium | 0.003 | 0.023 | 0.183 |
| Alloprevotella | 0.018 | 0.010 | 0.527* |
| Parasutterella | ＜0.001 | 0.007 | 0.025* |

Asterisks denote the difference is significant between groups (**P* < 0.05).

Mann-Whitney U Test was used to analyze the data.
