# Supplementary material for: Correlation between uterine microbiota and pregnancy outcomes of embryo transfer in overweight and obese women
Source: Front Cell Infect Microbiol. 2025 Feb 3;15:1515563. doi: 10.3389/fcimb.2025.1515563 (PMC11830673; doi:10.3389/fcimb.2025.1515563)
Supplement: Supplementary file 3 [file DataSheet3.docx]

**Supplementary Material: Detailed Endometrial Treatment Regimen**

1. Natural Cycle:
   Around day 12 of the menstrual cycle, vaginal ultrasound monitoring of the follicle is performed, alongside the measurement of peripheral blood female hormones to assess follicular and endometrial development. Ovulation time is determined, and after ovulation, 30 mg/day of dydrogesterone (Duphaston, Abbott) is administered orally. When the ultrasound indicates an endometrial thickness of ≥8 mm, a follicle diameter of 18-20 mm, and estradiol levels >0.65 nmol/L, embryo transfer is carried out on day 3 post-ovulation for cleavage-stage embryos or on day 5 or 6 post-ovulation for blastocyst-stage embryos.
2. Hormone Replacement Cycle:
   Starting on the day 2 of the menstrual cycle, patients take oral estradiol (Complex Packing Estradiol Tablets/Estradiol and Dydrogesterone Red Tablets, Abbott), with 4 mg/day for the Con group and 6 mg/day for the OwOb group, for a total of 12 days. On day 13 of estradiol treatment, a vaginal ultrasound is performed to assess the endometrial thickness. When the endometrial thickness reaches ≥8 mm, patients are switched to 4 mg estradiol + 20 mg dydrogesterone/day (Complex Packing Estradiol Tablets/Estradiol and Dydrogesterone Yellow Tablets, Abbott). Simultaneously, progesterone sustained-release vaginal gel (Crinone, Merck) is applied at 90 mg/day in the morning to transform the endometrium into the secretory phase. Taking the day when progesterone is used as day 0. Embryo transfer is performed on day 3 for cleavage-stage embryos, or day 5 or 6 for blastocyst-stage embryos.
3. Ovarian Stimulation Cycle:
   On day 3 of the menstrual cycle, patients start oral letrozole (Femara, Novartis), with 2.5 mg/day for the Con group and 5 mg/day for the OwOb group, for a total of 5 days. When the follicle reaches a diameter of ≥18 mm, a 5000 IU injection of chorionic gonadotrophin for injection (Chorionic Gonadotrophin for Injection, Livzon) is administered to induce ovulation. Embryo transfer is performed on day 3 post-ovulation for cleavage-stage embryos, or on day day 5 or 6 post-ovulation for blastocyst-stage embryos.
